# Nationwide Trends in Hospitalizations and Outcomes of Pulmonary Circulation Disorders Among Patients With Cannabis Use Disorder in the United States

**DOI:** 10.7759/cureus.22897

**Published:** 2022-03-06

**Authors:** Akhil Jain, Zainab Gandhi, Rupak Desai, Uvesh Mansuri, Bisharah Rizvi, Melissa Alvarez, Puneet Gupta

**Affiliations:** 1 Internal Medicine, Mercy Catholic Medical Center, Darby, USA; 2 Internal Medicine, Geisinger Wyoming Valley Medical Center, Wilkes Barre, USA; 3 Outcomes Research, Independent Researcher, Atlanta, USA; 4 Critical Care Medicine, Medstar Good Samaritan Hospital, Baltimore, USA; 5 Internal Medicine, Saint Agnes Medical Center, Fresno, USA; 6 Cardiology, Danbury Hospital, Danbury, USA; 7 Cardiology, Baptist Hospital, Madisonville, USA

**Keywords:** in-hospital outcomes, pulmonary hypertension, pulmonary circulation disorder, marijuana, cannabis

## Abstract

Background and objective

The use of cannabis through smoking and vaping has increased significantly over the past decade. However, the prevalence of pulmonary circulation disorder (PCD)-related hospitalizations among cannabis users and their outcomes remain poorly understood. In this study, we used a nationally representative sample to assess the prevalence and trends of hospitalization among cannabis users with PCD.

Methods

The National Inpatient Sample (NIS) datasets (2007-2014) were used to analyze hospitalizations of patients with cannabis user disorder with PCD (C-PCD arm) versus those without PCD (C-non-PCD arm) to ascertain demographics, comorbidities, and in-hospital outcomes including all-cause mortality and healthcare resource utilization.

Results

A total of 3,307,310 hospitalizations involving cannabis users were reported, of which 20,328 (0.61%) were related to PCD. We noted a 200% relative increase in hospitalizations in the C-PCD arm (linearly increasing from 0.3% to 0.9% from 2007 to 2014, p_trend_<0.001). When compared to the C-non-PCD arm, patients in the C-PCD arm tended to be older (mean age: 47 vs. 34 years), predominantly males (65.6% vs. 62.9%), with significantly higher rates of congestive heart failure (CHF, 28.8%), hypertension (HTN, 22%), chronic obstructive pulmonary disease (COPD, 21.5%), deficiency anemia (19.4%), and valvular heart disease (17.7%). The C-PCD arm had a statistically higher proportion of tobacco and amphetamine abusers (p<0.01) while the C-non-PCD arm had more cocaine and alcohol abusers (p<0.01). Urban teaching hospital admissions were more commonly associated with the PCD arm than the non-PCD arm (65.4% vs. 56.9%). In terms of hospital resource utilization, patients in the C-PCD arm had higher median hospital stay (six vs. three days) and more frequent discharges to a skilled nursing facility or home healthcare than the C-non-PCD group. All-cause mortality during hospitalization was found to be much higher in the C-PCD arm than the C-non-PCD arm (4.1% vs. 0.5%, p<0.001). Multivariable analysis revealed a two-fold higher risk for all-cause mortality with an adjusted odds ratio (OR) of 2.17 (95% CI: 1.99-2.36, p<0.001) with PCD.

Conclusion

The findings of this nationwide study revealed significantly increased rates of hospitalizations among cannabis users with PCD with two times higher odds of all-cause in-hospital mortality. Further prospective studies are warranted in this subgroup of patients to confirm these findings and facilitate the management of these patients.

## Introduction

Recreational cannabis use is becoming more prevalent in the United States with the legalization/decriminalization efforts in various states. Various physiological and pathological effects related to its use are also increasingly being recognized. Particulate matter, reactive oxygen species, and polycyclic aromatic hydrocarbons from cannabis smoking are associated with 20 times more intense inflammatory responses than tobacco smoking [1-2]. Daily use of cannabis has been linked to chronic coughing and wheezing in recently published studies [3,4]. The effects of cannabis on the pulmonary vasculature are poorly understood. In our study, we examined the prevalence and trends of hospitalization among cannabis users with pulmonary circulation disorders (PCDs) using a nationally representative sample.

We previously presented our study findings at the American College of Cardiology's (ACC) 70th Annual Scientific Session & Expo in 2021.

## Materials and methods

We conducted a retrospective analysis using Healthcare Cost and Utilization Project-provided National Inpatient Sample (NIS) datasets (2007-2014) (https://www.hcup-us.ahrq.gov/nisoverview.jsp). Statistically anonymized yearly discharge data was derived from a 20% stratified sample of discharges from community hospitals (excluding rehabilitation and long-term acute care hospitals) representing >95% of the US population. Similar to prior studies, we used publicly accessible NIS datasets. Since the data are de-identified, we did not require approval from the Institutional Review Board.

We identified hospitalizations among patients with cannabis use disorder using the relevant International Classification of Diseases, Ninth Revision, Clinical Modification (ICD-9 CM) codes (304.30, 304.31, 304.32, 305.20, 305.21, and 305.22). We identified admissions for PCD using ICD-9 CM codes 415.11-415.19, 416.0-416.9, and 417.9. The study included cannabis users with any primary or secondary discharge diagnoses of PCDs. Other comorbidities considered in the study were identified from secondary discharge diagnoses. “C-PCD” and “C-non-PCD” abbreviations are used to refer to hospitalizations with cannabis use disorder that is PCD-related and cannabis use disorder without PCD, respectively.

The primary outcomes were the frequency and trends in admissions for PCD with respect to age, gender, and racial differences, and odds of subsequent inpatient mortality among patients with cannabis use disorder from 2007 to 2014. Secondary outcomes included comorbidities, the median length of stay (days), and hospitalization charges. Data on national trends were analyzed using sampling weights and the linear-by-linear association test in the SPSS Statistics software V24.0 (IBM Corp., Armonk, NY). The relative increase in the prevalence of PCD among cannabis users was calculated by comparing the prevalence in 2014 vs. 2007. Multivariable regression analysis was performed to assess the odds of in-hospital mortality with PCD by adjusting for potential confounders including sociodemographics, hospitalization attributes, payer status, median household income quartile, and pre-existing cardiovascular and extra-cardiac comorbidities including concomitant substance abuse. A two-tailed p<0.05 was considered the statistically significant threshold.

## Results

**Figure 1:**
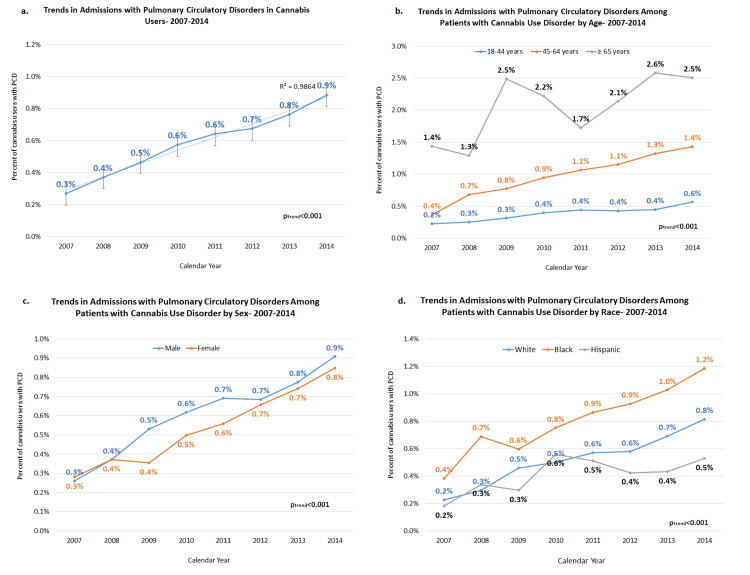
Trends in admissions related to pulmonary circulation disorders among patients with cannabis use disorder (2007-2014) a. Overall trends in admissions. b. Trends in admissions by age. c. Trends in admissions by sex. d. Trends in admissions by race

From 2007 to 2014, there were 3,307,310 cannabis use disorder-related hospitalizations nationwide. Hospitalizations for C-PCD accounted for 20,328 (0.6%) of these cases. Overall, we observed a 200% relative increase in C-PCD hospitalizations (linearly increasing from 0.3% to 0.9% over the period, p_trend_<0.001, Figure 1a). Among patients with cannabis use disorder, the geriatric age group (≥65 years) compared to younger patients (Figure 1b), males compared to females (Figure 1c), and black patients compared to white or Hispanic patients (Figure 1d) demonstrated more prominently rising trends in PCD-related admissions. Baseline demographics and comorbidities were compared between the two groups: C-PCD and C-non-PCD (Table 1). The C-PCD cohort was relatively older compared to the C-non-PCD cohort (median age of 47 years vs. 34 years) with a higher proportion of males (65.6% vs. 62.9%), and black patients (41.9% vs. 31.3%) compared to the C-non-PCD cohort (p<0.05). Comorbidities, including congestive heart failure (CHF), hypertension (HTN), previous myocardial infarction, valvular heart disease, chronic obstructive pulmonary disease (COPD), peripheral vascular disease, diabetes with or without complications, dyslipidemia, coagulopathy, hypothyroidism, liver disease, renal failure, anemia, obesity, and malignancy with or without metastasis, were significantly higher in the C-PCD arm (p<0.001). Absolute differences in comorbidities were noteworthy for CHF (28.8%), HTN (22%), COPD (21.5%), deficiency anemia (19.4%), and valvular disease (17.7%). C-PCD arm participants were significantly more likely to abuse tobacco and amphetamine, while C-non-PCD arm participants were more likely to abuse alcohol and cocaine (p<0.001).

**Table 1:** Baseline characteristics of hospitalizations among patients with cannabis use disorder with pulmonary circulation disorder vs. those without P<0.05 indicates statistical significance. All p<0.001 except for cocaine abuse (p=0.052) PCD: pulmonary circulation disorder; IQR: interquartile range; HMO: health maintenance organization; MI: myocardial infarction, PCI: percutaneous coronary intervention; CABG: coronary artery bypass grafting; SNF: skilled nursing facility; ICF: intermediate care facility

Characteristics			Non-PCD (n=3,286,982)	PCD (n=20,328)	Overall cannabis use disorder (n=3,307,310)
Age at admission, years, median (IQR)			34 (25-47)	47 (33-55)	34 (25-47)
Sex	Male	62.90%		65.60%	62.90%
	Female	37.10%		34.40%	37.10%
Race	White	54.50%		47.50%	54.40%
	African American	31.30%		41.90%	31.40%
	Hispanic	9.20%		6.20%	9.20%
	Asian or Pacific Islander	0.80%		0.80%	0.80%
	Native American	1.10%		1.20%	1.10%
	Others	3.10%		2.40%	3.10%
Non-elective admission		87.20%		94.60%	87.20%
Primary expected payer	Medicare	15.50%		29.70%	15.60%
	Medicaid	36.90%		36.60%	36.90%
	Private including HMO	20.90%		14.40%	20.90%
	Self-pay/no charges/others	26.70%		19.30%	26.70%
Median household income national quartile for patient ZIP code	0-25th	41.60%		46.90%	41.60%
	76-100th	13.10%		10.90%	13.10%
Location/teaching status of the hospital	Rural	9.40%		6.20%	9.40%
	Urban non-teaching	33.70%		28.30%	33.70%
	Urban teaching	56.90%		65.40%	56.90%
Region of hospital	Northeast	21.90%		13.50%	21.80%
	Midwest	26.70%		27.70%	26.70%
	South	33.50%		34.60%	33.50%
	West	18.00%		24.20%	18.00%
Comorbidities					
Alcohol abuse		26.70%		23.50%	26.60%
Deficiency anemias		8.70%		28.10%	8.80%
Rheumatoid arthritis/collagen vascular diseases		1.00%		2.90%	1.00%
Congestive heart failure		1.90%		30.70%	2.10%
Chronic pulmonary disease		16.00%		37.50%	16.10%
Coagulopathy		2.90%		12.60%	2.90%
Depression		10.90%		15.20%	11.00%
Diabetes, uncomplicated		7.10%		14.80%	7.20%
Diabetes with chronic complications		1.80%		4.80%	1.80%
Hypertension		24.70%		46.70%	24.90%
Hypothyroidism		2.90%		5.30%	2.90%
Liver disease		4.10%		10.90%	4.10%
Fluid and electrolyte disorders		16.90%		41.30%	17.00%
Metastatic cancer		0.40%		2.10%	0.50%
Obesity		7.30%		19.30%	7.40%
Peripheral vascular disorders		1.50%		5.30%	1.50%
Renal failure		3.10%		16.10%	3.10%
Solid tumor without metastasis		0.50%		1.60%	0.50%
Valvular disease		0.90%		18.60%	1.00%
Dyslipidemia		9.60%		17.80%	9.60%
Previous MI/PCI/CABG		3.30%		8.50%	3.40%
Smoking		53.60%		66.60%	53.60%
Cocaine abuse		19.80%		19.30%	19.80%
Amphetamine abuse		6.30%		7.50%	6.30%
In-hospital outcomes					
All-cause mortality		0.50%		4.10%	0.50%
Disposition of patient	Routine	82.30%		62.90%	82.20%
	Transfer to short-term hospital	1.90%		3.70%	1.90%
	Other transfers (SNF, ICF, etc)	7.10%		12.50%	7.10%
	Home healthcare	3.20%		11.50%	3.20%
Length of stay, days, median (IQR)			3 (2-6)	6 (3-10)	3 (2-6)
Total charges, USD, median (IQR)			14,155 (7,962-27,256)	37,557 (18,872-78,980)	14,224 (7,987-27,458)

Four regions were identified as hospitalization areas: the Northeast (NE), Midwest (MW), South (S), and West (W). Hospitalizations related to cannabis use disorder were highest in the South, followed by MW, NE, and the West. The South region had almost twice as many hospitalizations as the West region (33.5 vs. 18%). Hospitalizations were quite similar in the South and MW-NE in both groups; in the West, there were more C-PCD admissions (24.2% vs. 18.0%). Urban teaching hospitals had more C-PCD admissions than C-non-PCD (65.4% vs. 56.9%); non-teaching and rural hospitals had more C-non-PCD admissions. Large hospitals admitted more patients from both arms than medium and small hospitals (62.9% vs. 37.1%). Across both arms, non-elective hospitalizations were substantially greater than elective ones (overall 87.2% vs. 12.8%). C-PCD admissions had a higher average length of stay than C-non-PCD (six vs. three days). Routine discharges to home or self-care were 20% higher in the C-non-PCD arm, while transfers to short-term or intermediate care facilities, skilled nursing facilities, and home healthcare were higher in the C-PCD arm. The most common primary expected payer was Medicaid (around 36%) in both the groups followed by other payers. A significantly higher proportion of all-cause mortality (4.1% and 0.5%) was observed during hospitalization among the C-PCD cohort than the C-non-PCD cohort with adjusted multivariable analysis confirming approximately two times higher odds of inpatient mortality, the adjusted odds ratio of 2.17, and 95% confidence interval of 1.99-2.36 (p<0.001) with PCD.

## Discussion

To the best of our knowledge, this is the first study to assess the prevalence and trends of PCD among patients with cannabis use disorder. There is conflicting evidence regarding the effects of cannabis on pulmonary vasculature. Vasodilation via endocannabinoids-activated CB1 and G protein-coupled receptors on the vasculature vasodilation [5,6] versus hypoxic pulmonary vasoconstriction via intracellular enzymatic fatty acid amide hydrolase (FAAH) or monoacylglycerol lipase (MAGL) metabolization [5,7,8] or COX and LOX pathways [9,10] have been reported. In-vitro mice studies have shown a higher expression of FAAH in the lungs [11]. These are the short-term effects of cannabis.

Our study showed that the PCD arm often consisted of male and black populations with cannabis use disorder. Studies have suggested that men and blacks are more likely to use cannabis recreationally and for longer periods, which may increase their risk of developing the long-term pathophysiological effects of cannabis [12,13]. Patients with PCD who used cannabis were more likely to have comorbidities such as CHF, HTN, COPD, heart disease, obesity, and other conditions that were associated with PCD. Lang and Palazzini in their review discussed the effect of comorbidities in the diagnosis and management of pulmonary arterial hypertension [14]. Cardiovascular comorbidities including traditional risk factors, coagulopathy, hypothyroidism, liver disease, renal failure, anemia, and malignancy with or without metastasis were significantly higher in the C-PCD arm compared to the C-non-PCD arm, which also hints at a potential link between the underlying multi-system association of cannabis use disorder and PCD.

As part of our analysis, we also assessed how PCD among cannabis users impacts the healthcare system. We found that cannabis users with PCD were more likely to require emergent hospitalizations (94.6% vs. 87.2%, p<0.001), often at large urban academic hospitals, and tend to stay longer as inpatients. Heresi et al. reported higher all-cause resource utilization and annual healthcare costs in patients with pulmonary HTN, and 35.4% of the total cost was driven by inpatient admissions [15]. In our study, we found that cannabis users with PCD had two times higher odds of in-hospital mortality than cannabis users without PCD. A nationwide cohort study from Taiwan showed higher mortality for patients with pulmonary HTN than controls (56.45 vs. 18.51 per 1,000 person-years, p<0.0001). The long-term survival rates of these patients at one, five, and 10 years were 87.9%, 72.5%, and 62.6%, respectively, which were significantly lower than controls with 98.4%, 90.8%, and 83.6% at one, five, and 10 years, respectively [16]. Additionally, Mehari et al. described national trends for all pulmonary HTN-related deaths and hospitalizations during 1999-2008 and concluded that pulmonary HTN-related mortality and hospitalization numbers and rates increased from 1999 to 2008 [17].

The most significant finding of our study is the 200% relative increase in PCD-related hospitalization rates among cannabis users from 2007 to 2014. Although cannabis use has increased three times in the last decade [18], improved diagnostic modalities with more awareness among healthcare providers may have contributed to this finding. These results point towards the need for more studies to better understand the mechanism of PCD associated with cannabis use for the development and application of therapeutic agents for proper management and minimizing the utilization of hospital resources.

This retrospective cross-sectional study has some limitations. We relied on administrative claims data, which could have included coding errors related to diagnoses and comorbidities. PCD was used as an umbrella term for all pulmonary circulatory disorders without any subgrouping as per WHO classification. Also, comorbid conditions may have been underreported. The database did not report follow-up data to gauge morbidity and mortality after discharge. Additionally, we did not have diagnostic data to gauge the screening tools. Owing to its retrospective design without a prospectively controlled sample selection, this study could not report any causative role of cannabis use in PCD or subsequent outcomes. Furthermore, the NIS dataset did not reveal the dose, duration, and mode of cannabis use. Nevertheless, this database offers a large-scale nationally representative sample of admissions for performing epidemiological and outcomes research on this understudied subject among patients with cannabis use disorder.

## Conclusions

This population-based analysis has demonstrated the alarmingly rising trends in PCD-related hospitalizations among patients with cannabis use disorder, which is associated with a two-fold increase in in-hospital mortality when controlled for confounders, between 2007 and 2014. Our study provides valuable insight into the under-researched area of PCD in cannabis users and paves the way for further human studies to strengthen the association and better understand the mechanism of PCD in cannabis users, which may facilitate the development of better management protocols and improved long-term outcomes for these patients.
